# Cytotoxicity and Inflammatory Effects of Chitin Nanofibrils Isolated from Fungi

**DOI:** 10.1021/acs.biomac.3c00710

**Published:** 2023-11-21

**Authors:** Aitor Larrañaga, Carlos Bello-Álvarez, Erlantz Lizundia

**Affiliations:** † Department of Mining-Metallurgy Engineering and Materials Science, POLYMAT, Faculty of Engineering in Bilbao. University of the Basque Country (UPV/EHU), Plaza Ingeniero Torres Quevedo 1, 48013 Bilbao, Biscay, Spain; ‡ Life Cycle Thinking Group, Department of Graphic Design and Engineering Projects. University of the Basque Country (UPV/EHU), Plaza Ingeniero Torres Quevedo 1, 48013 Bilbao, Biscay, Spain; § BCMaterials, Basque Center for Materials, Applications and Nanostructures, Edif. Martina Casiano, Pl. 3 Parque Científico UPV/EHU Barrio Sarriena, 48940 Leioa, Biscay, Spain

## Abstract

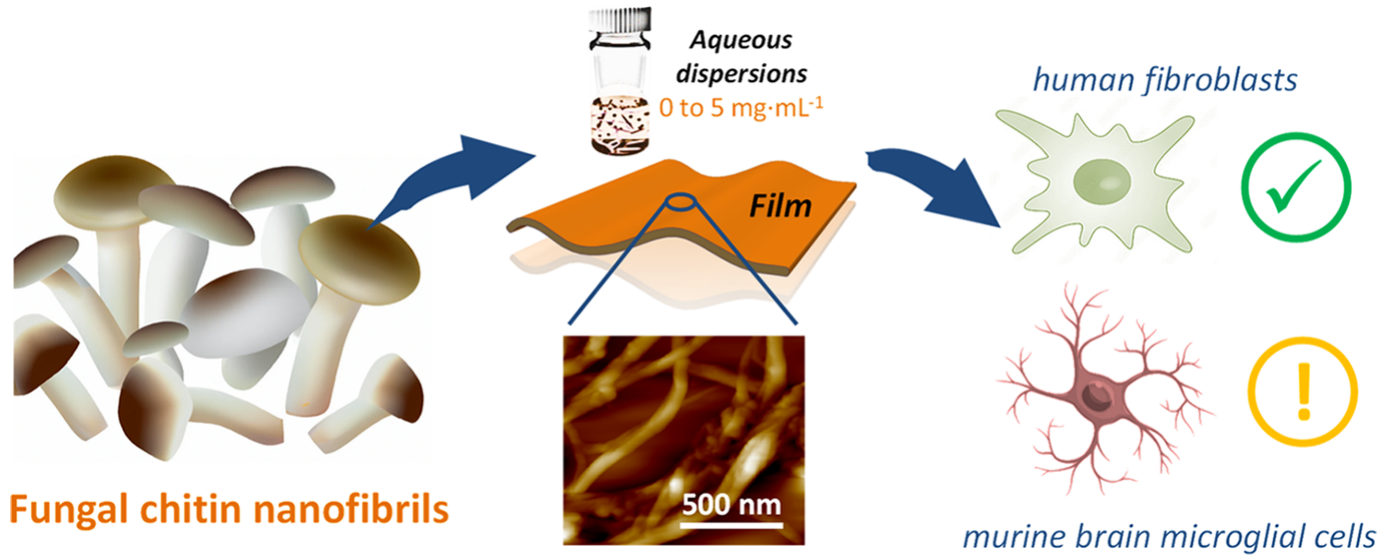

Fungal nanochitin can assist the transition from the linear fossil-based economy to a circular biobased economy given its environmental benefits over conventional crustacean-nanochitin. Its real-world implementation requires carefully assessing its toxicity so that unwanted human health and environmental issues are avoided. Accordingly, the cytotoxicity and inflammatory effects of chitin nanofibrils (ChNFs) from white mushroom is assessed. ChNFs are few nanometers in diameter, with a 75.8% *N*-acetylation degree, a crystallinity of 59.1%, and present a 44:56 chitin/glucan weight ratio. Studies are conducted for aqueous colloidal ChNF dispersions (0–5 mg·mL^–1^) and free-standing films having physically entangled ChNFs. Aqueous dispersions of chitin nanocrystals (ChNCs) isolated via hydrochloric acid hydrolysis of α-chitin powder are also evaluated for comparison. Cytotoxicity studies conducted in human fibroblasts (MRC-5 cells) and murine brain microglia (BV-2 cells) reveal a comparatively safer behavior over related biobased nanomaterials. However, a strong inflammatory response was observed when BV-2 cells were cultured in the presence of colloidal ChNFs. These novel cytotoxicity and inflammatory studies shed light on the potential of fungal ChNFs for biomedical applications.

## Introduction

The use of fossil-fuel based materials is driving our society toward an unprecedented climate crisis with notable environmental issues related to raw material scarcity,^1^ large global footprint,^2^ declining fossil resource availability,^1^ and uncontrolled accumulation of plastic waste in terrestrial, river, or marine ecosystems.^3^ Transitioning toward a biobased economy could partially address these global challenges given the inherent renewability and biodegradability of materials from biological origin.^4^ In this context, a great deal of attention is being paid to the exploitation of natural biopolymers that are mainly composed of a few building blocks containing carbon and originating from the cells of living organisms such as plants or microorganisms. Generally, natural biopolymers fulfill the requisites of low cost, wide and local availability, processability, thermal and mechanical performance, and ease of chemical modification.^5,6^ Developing (nano)fibrillated biopolymers opens new horizons toward new transformative applications with multiple functions, where the mechanical, optical, thermal, and ionic properties are above the properties shown by the parent material.^7^ Diverse biopolymer fibers can be separated into fibrils of decreasing diameter (ranging from a few microns to a few nanometers) that are ultimately composed of ordered linear molecular chains. Among those, cellulosic biocolloids (colloidal entities composed of cellulose and its derivatives), in the form of cellulose nanocrystals (CNCs) or cellulose nanofibrils (CNFs), show a dominant position at both fundamental and applied research, with ease of chemical modification^8^ and high technology readiness levels (TRLs).^7,9,10^ Cellulose is decomposed into nontoxic glucosidic chains, enabling its use in many environmental and biomedical applications.^11^

However, there are other potential biopolymers open to exploration given the vast library of biobased materials that nature offers. With a similar structure to cellulose, chitin has also been deconstructed to obtain chitin nanocrystals (ChNCs) and chitin nanofibrils (ChNFs).^12^ Chitin contains ∼6 wt % nitrogen from acetamide groups and it is found in many living organisms; i.e., as a major structural component in the exoskeletons (shells) of arthropods, insects, or fungi.^13^ Colloidal chitin extracted from crustaceans has shown important applications in photonic devices,^14^ energy storage,^15^ or barrier applications in films.^16^ However, the isolation of these nanoparticles from crustaceans, where it appears together with CaCO_3_, proteins and minerals, requires harsh chemical and/or mechanical treatments, often involving strong acid hydrolysis and/or chemical oxidation steps for demineralization, deproteinization, bleaching, or fibrillation.^13^ These processes increase the environmental impacts of resulting biocolloids, jeopardizing their implementation as sustainable materials. On the contrary, the chitin in fungal resources does not coexist with CaCO_3_. As a result, colloidal chitin can be easily isolated from fungi under mild conditions. As a result, fungal ChNF isolation shows a lower global warming potential (18.5 kg CO_2_-equiv·kg^–1^) when compared with the 543.5 or 906.8 kg CO_2_-equiv·kg^–1^ generated upon conventional chitin nanocrystal extraction from crab or shrimp shells, respectively.^17^

These merits position fungal ChNFs at the forefront of research toward environmentally sustainable materials. In a pioneering work, Nawawi et al. reported in 2019 a very simple procedure to isolate chitin nanofibers from the white mushroom (*Agaricus bisporus*), where solely a short mechanical agitation in a kitchen blender and a mild alkaline treatment were needed.^18^ This material was then processed in the form of nanopapers with tensile strengths above 200 MPa^18^ and has been used for the ultrafiltration of organic solvents and water^19^ or as battery electrolytes in the form of gels.^20^ However, the safety of these materials remains a question to be answered to pave their way into real-world applications and ensure that these materials are not hazardous for both human health and the environment.^20^ In this sense, although CNCs and CNFs, cellulose analogues to ChNFs, have, in general, demonstrated negligible-to-low (cyto)toxicity,^21,22^ pulmonary inflammation for CNFs has been seen, while chemical modification impairs low cytotoxicity to CNFs.^23−25^ The size and morphology, crystallinity degree, or surface chemistry are key aspects in determining the nanotoxicity of nanocelluloses. For example, CNCs and CNFs show a length-dependent mechanisms of toxicity on liver cells, where short nanoparticles triggered significant cytotoxicity in Kupffer cells.^26^

In this context, *in vitro* toxicity studies of ChNFs are of particular relevance as nanomaterials in general have an increased ability to migrate to various organs and tissues and cross physiological barriers.^27^ This would help to foresee any potential toxic effect of chitin biocolloids from fungi induced by inhalation, dermal exposure, or other routes of administration. In this context, human fibroblasts are one of the preferred cells to study the cytotoxicity aspects related to biobased colloids since they are well accepted by the ISO/EN 10993 procedures for the biological evaluation of biomedical devices.^28^ In addition, microglia are a relevant type of cells involved in the regulation of neuroinflammatory responses and immune surveillance.^29^ These cells have proven useful to determine the nanotoxicity of nanomaterials such as silver,^30^ or titanium dioxide.^31^ Therefore, studies on microglia could help to exclude undesired pro-inflammatory effects induced by ChNFs.

Accordingly, here we isolated ChNFs from white mushrooms to validate their nontoxicity and open the use of fungal biocolloids into real-world applications. ChNFs have a crystallinity degree of 59.1% and are composed of chitin and amorphous glucans (44 wt % chitin), and the chitin fraction has a *N*-acetylation degree of 75.8%. Studies are conducted on colloidally stable ChNF dispersion and free-standing nanopaper films using human fibroblasts (MRC-5 cells) and murine brain microglia (BV-2 cells). No significant differences on the metabolic activity of MRC-5 and BV-2 cells were observed, even at large exposure doses, indicating ChNFs from fungal resources are comparatively safer than CNCs or CNFs. Given the lack of toxicity of these materials and their inherent properties, we envisage the implementation of chitin nanofibrils not only in water remediation or energy storage, but also in biomedical applications.

## Experimental Section

### 2.1 Materials

White mushrooms (*Agaricus bisporus*) purchased from a local store in Bilbao (Spain) were used for chitin nanofibril isolation. Chitin from shrimp shells (practical grade powder) was purchased from Sigma-Aldrich. Sodium hydroxide pellets (NaOH, ≥97%) and hydrochloric acid (HCl, 37%) were obtained from Honeywell Fluka. Human lung fibroblasts (MRC-5, CCL-171) were acquired from ATCC (U.S.A.), whereas murine microglia (BV-2) were acquired from AcceGen Biotech (U.S.A.). Dulbecco’s modified Eagle medium (DMEM), Hanks’ balanced salt solution (HBSS), penicillin–streptomycin (P/S), fetal bovine serum (FBS), AlamarBlue cell viability reagent, and rhodamine-phalloidin were supplied by Fisher Scientific (Spain). Fluoroshield with DAPI, Triton X-100, Tween 20, and lipopolysaccharides (LPS) were supplied by Sigma-Aldrich (Spain), and 16% formaldehyde solution and the Griess Reagent were supplied by ThermoFisher Scientific (Spain). Mouse TNF-alpha DuoSet Elisa was purchased from Biotechne (U.K.).

### 2.2 Chitin Nanofibril (ChNF) and Chitin Nanocrystal (ChNC) Isolation and Film Fabrication

Colloidal chitin nanofibrils (ChNFs) were isolated from white mushrooms following a top-down approach.^18^ Mushrooms were frozen at −10 °C for 1 week just after purchase. For biocolloid isolation, 500 g of frozen white mushrooms were immersed in 1 L of distilled water during 5 min and were manually washed to remove any dirt. Then, the mushrooms were blended for 5 min (Power Black Titanium 1800) and the obtained slurry was heated at 85 °C for 30 min in a three-neck round-bottom flask. The mixture was then washed by filtration, and the cake was recovered and redispersed again in distilled water for an additional treatment in 1 M NaOH at 65 °C for 180 min under magnetic stirring. After being washed by filtration, the mixture was blended again for 1 min. Finally, the biocolloids were stored at 1.0 wt % (aqueous dispersion) at 4 °C until use.

Chitin nanocrystals (ChNCs) were prepared via acid hydrolysis of neat α-chitin powder.^32^ An amount of 1:15 (w/v) commercial pure chitin powder from shrimp was added to a 3 M HCl solution and was magnetically stirred at 85 °C for 90 min. The reaction was quenched by adding a 3-fold ice-cold water quantity. The HCl was removed by three centrifugation steps (8000 g for 10 min at 25 °C); the supernatant was discarded, and the pellet was resuspended in distilled water. A dispersion of ∼100 mL was sonicated using a UP400 St sonicator (Hielscher) equipped with a S24d14D sonotrode at a power of 200 W for 8 min while cooled on ice. Subsequently, a centrifugation step at 8000 g was applied for 10 min at 25 °C, and the supernatant containing the ChNCs was collected. For further purification, the dispersion was dialyzed against distilled water for a week using regenerated cellulose dialysis tubing having a molecular weight cut off (MWCO) of 12–14 kDa (Medicell Membranes Ltd.). The ChNC concentration was obtained upon drying ∼3 g of suspension and measuring the dry content. ChNCs were stored at 4 °C at a concentration of 3.5 wt %.

ChNF films were prepared by solvent casting. Hot pressing was not used to avoid undesired surface patterning effects by the molding. Briefly, 8 mL of 1 wt % aqueous ChNF dispersion was casted onto polystyrene weighing dishes (60 × 60 mm) and was allowed to evaporate for 96 h at 20 °C. The resulting film was further dried in an oven at 50 °C for 24 h.

### 2.3 Physico-Mechanical Characterization

Atomic force microscopy (AFM) observations were conducted using a Veeco Instrument’s MultiMode SPM 004-130-000 AFM at room temperature. For biocolloid observation, a ChNF suspension droplet at a concentration of 0.02 mg·mL^–1^ was coated onto a mica substrate, and water was allowed to evaporate at room temperature. For film characterization, the solvent-casted film was directly mounted onto a stainless steel AFM holder, and the film was observed. The NanoScope Analysis 1.9 program was employed to analyze the recorded images. ChNFs and ChNCs were observed by transmission electron microscopy (TEM) on a JEOL JEM 1400 Plus apparatus at an acceleration voltage of 100 kV. A 3 μL droplet (0.01 wt % aqueous dispersion) was deposited onto a hydrophilic EMS CF300-Cu grid (glow discharge treatment; 10 mA during 30 s in a Leica EM ACE200) and the biocolloids were negatively stained with 1% uranyl acetate (UO_2_(CH_3_COO)_2_) for 20 s (the uranyl acetate was then removed with a filter paper).

Attenuated total reflectance Fourier transform infrared (ATR-FTIR) spectra were obtained by using a Jasco FT/IR-6100 spectrometer (ATR optics; 2 cm^–1^ resolution). Room temperature X-ray diffraction (XRD) was conducted in a PHILIPS X’PERT PRO automatic diffractometer in theta–theta configuration, secondary monochromator with Cu–Kα radiation (λ = 1.5418 Å) and a PIXcel solid state detector. Carbon nuclear magnetic resonance (^13^C NMR) spectra were acquired in a Bruker Avance DPX 300 (Bruker, U.S.A.) at 75.5 MHz resonance frequency. Spectra were obtained at room temperature using 40 mg, inverse gated decoupled sequence, 3 s acquisition time, 4 s delay time, 5.5 μs pulse, spectral width 18800 Hz, and >10000 scans. Zeta-potential of water-dispersed ChNFs and ChNCs (0.02 mg·mL^–1^) for pH values ranging from 2 to 10 was obtained using a Malvern Zetasizer Nano-ZS. The pH was tuned upon addition of 0.1 M NaOH or 0.1 M HCl. The thermodegradation of ChNF films was assessed in a TGA METTLER TOLEDO 822e instrument using platinum pans at a heating rate of 10 °C·min^–1^ with a 50 mL·min^–1^ N_2_ flow.

The surface topology of the ChNF films was analyzed using a scanning probe microscope Dimension ICON from Bruker with NanoScope Analysis 1.9 software. Experiments were conducted in tapping mode with an integrated silicon tip/cantilever. Water was used as the probe liquid for the determination of the contact angle. Measurements were carried out by the sessile drop method (5 μL per drop) using a Krüss Drop Shape Analyzer DSA100 at room temperature. The average value was calculated by using four measurements. The tensile properties of the ChNF films were analyzed using a universal testing machine (MTC-100 from IDM) equipped with a 500 N load cell. Fifteen mm long, 5 mm wide, and 30 ± 2 μm films were used, with a deformation rate of 0.5 mm·min^–1^. Average and standard deviation values were determined over three measurements. Mercury intrusion porosimetry (POREMASTER-60 GT, Quantachrome Instruments, Inc.) was applied to measure the porosity of films at a maximum pressure of 241 MPa

### 2.4 *In Vitro* Studies

To study the potential cytotoxicity of the isolated chitin nanofibrils and chitin nanocrystals, the metabolic activity of MRC-5 and BV-2 cells was determined in the presence of increasing concentrations (0–5 mg·mL^–1^) of ChNFs and ChNCs. To avoid potential contamination, ChNFs and ChNCs were thoroughly washed with 70% ethanol prior to their incorporation in the cell culture media. Cells were seeded in a 96-well plate at a density of 5000 cells per well. After 24 h, the medium was aspirated and substituted by complete medium (DMEM + 10% FBS) containing increasing amounts of ChNFs and ChNCs. After 24 h, the metabolic activity of the cells was determined by the AlamarBlue assay. Additionally, the potential pro-inflammatory response of BV-2 cells to the presence of ChNFs and ChNCs was also evaluated. Accordingly, BV-2 cells were seeded in a 48-well plate at a density of 50,000 cells per well. After 24 h, media was aspirated and substituted by complete media containing increasing amounts of ChNFs and ChNCs (0, 0.1, 1, and 5 mg·mL^–1^). LPS at a concentration of 20 ng·mL^–1^ was used as a positive control. After 24 h, the media was collected, and the presence of nitrites was quantified by the Griess Reagent. The production of TNF-alpha in the media was determined by ELISA following the protocol provided by the supplier.

To evaluate the cytotoxicity of ChNF films and their capacity to allow cell adhesion and serve as scaffolds, circular samples of 6 mm diameter were first punched out from the free-standing films obtained upon solvent casting. These films were placed in 24-well plates, washed with 70% ethanol, and further sterilized by exposure to UV-light (30 min). Then, cells (MRC-5 or BV-2 cells) were seeded at a density of 10000 cells per sample. After 24 and 48 h, the metabolic activity of cells was determined by the AlamarBlue assay. At these time-points, cells were fixed with 4% paraformaldehyde solution and their cytoskeleton and nuclei were respectively stained with rhodamine-phalloidin and DAPI, as previously described by us.^33^ Cells were finally observed under an inverted fluorescent microscope (Nikon Eclipse Ts2, Nikon).

### 2.5 Statistical Analysis

In the *in vitro* studies, the results are presented as mean ± SD. One-way analysis of variance (ANOVA) was used to test the statistical differences between groups, with the Bonferroni post hoc test and a confidence level of 95% (*p* < 0.05).

## Results and Discussion

### *In Vitro* Evaluation of Dispersed Chitin Nanofibrils

**Figure 1:**
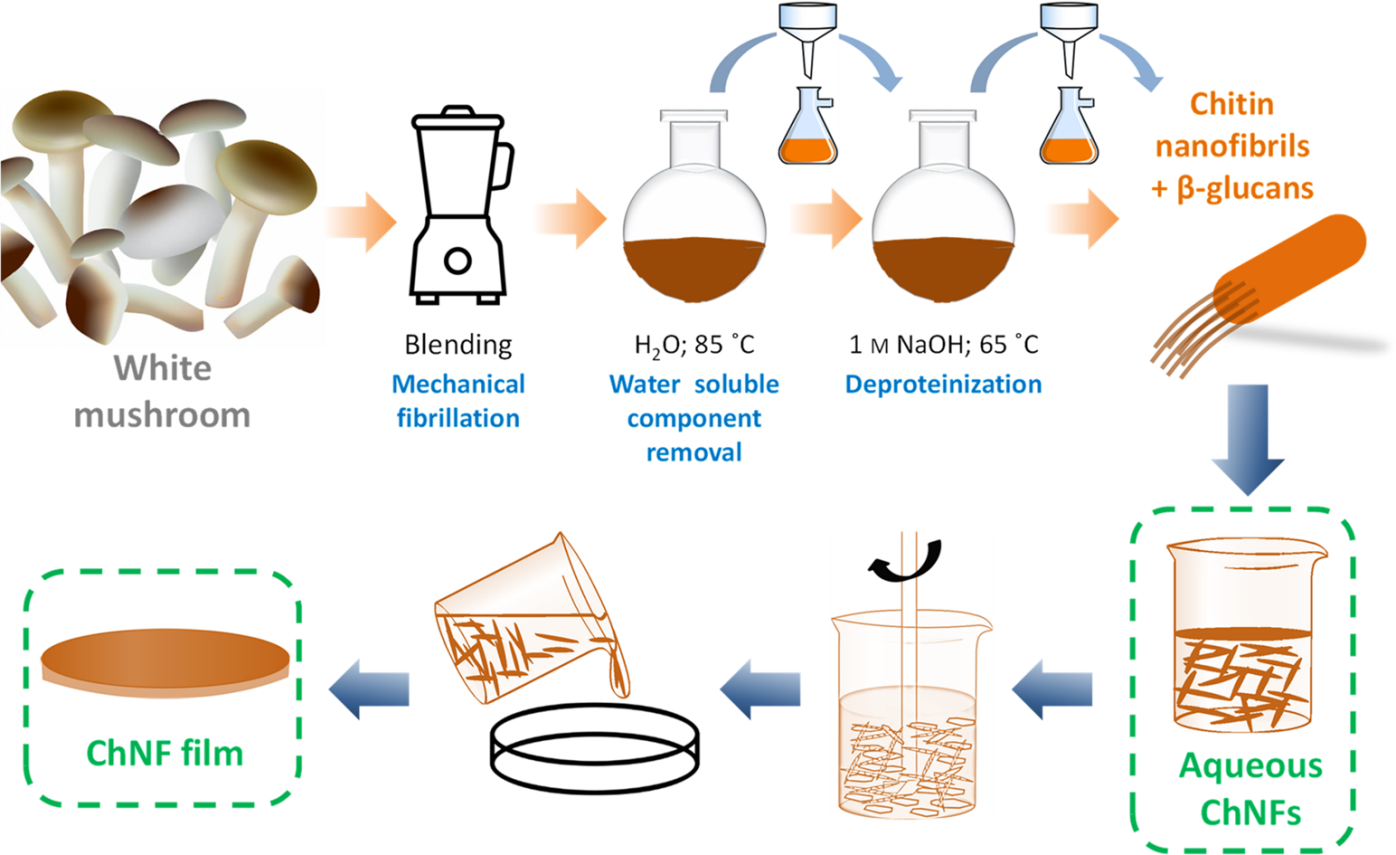
ChNF isolation from white mushroom (*Agaricus bisporus*) using mechanical blending and deproteinization. Toxicity studies are carried out on aqueous ChNF dispersions (at different concentrations) and on ChNF free-standing films, highlighted in green.

The aim of this work is to evaluate any potential cytotoxic and inflammatory response that ChNFs isolated from fungi may originate. To do so, our efforts focus on water-dispersed ChNFs and free-standing ChNF-based films (Figure 1, highlighted within the green box). A top-down approach is followed to isolate the native nanofibrils located at the inner cell wall of certain fungal species. These chitinous nanofibrils act as structural polymer embedded within a β-glucan matrix, providing mechanical stability to fungal cell walls.^13,34^ Colloidal chitin nanofibrils are obtained after an initial fibrillation process with a conventional cooking blender, then submitting the slurry to a hot-water treatment (85 °C for 30 min, it removes water-soluble components) and a final deproteinization process (1 M NaOH, 65 °C for 180 min) to remove proteins, lipids, and certain polysaccharides.^18^ Buchner filtration is applied to wash the extract soluble components. Finally, a 1 wt % colloidal dispersion of ChNFs in water is obtained. As indicated by the visual appearance of dispersions in Figure S1, ChNFs remain stable in water for days.^35^ This colloidal stability is of great importance when considering this nanomaterial as an injectable vehicle for biomedical applications (e.g., drug delivery).

This dispersion has also been used to prepare free-standing films by a simple solvent-casting. During this process, water slowly evaporates, and the concentration of the slurry increases so nanofibers physically entangle. As water continues to evaporate, capillary forces provide attraction between individual nanofibers.^36^ With further water evaporation, these fibers become close to each other, and secondary attraction forces such as hydrogen bonding occur between nanofibrils, which in turn yields free-standing films with remarkable mechanical properties.^7^ Thanks to the intricate nanoparticle entanglement, ChNF films do not redisperse when immersed in water (Figure S2), which is in contrast with free-standing films prepared upon simple solvent-casting of other biocolloids such as cellulose nanocrystals.^37^

**Figure 2:**
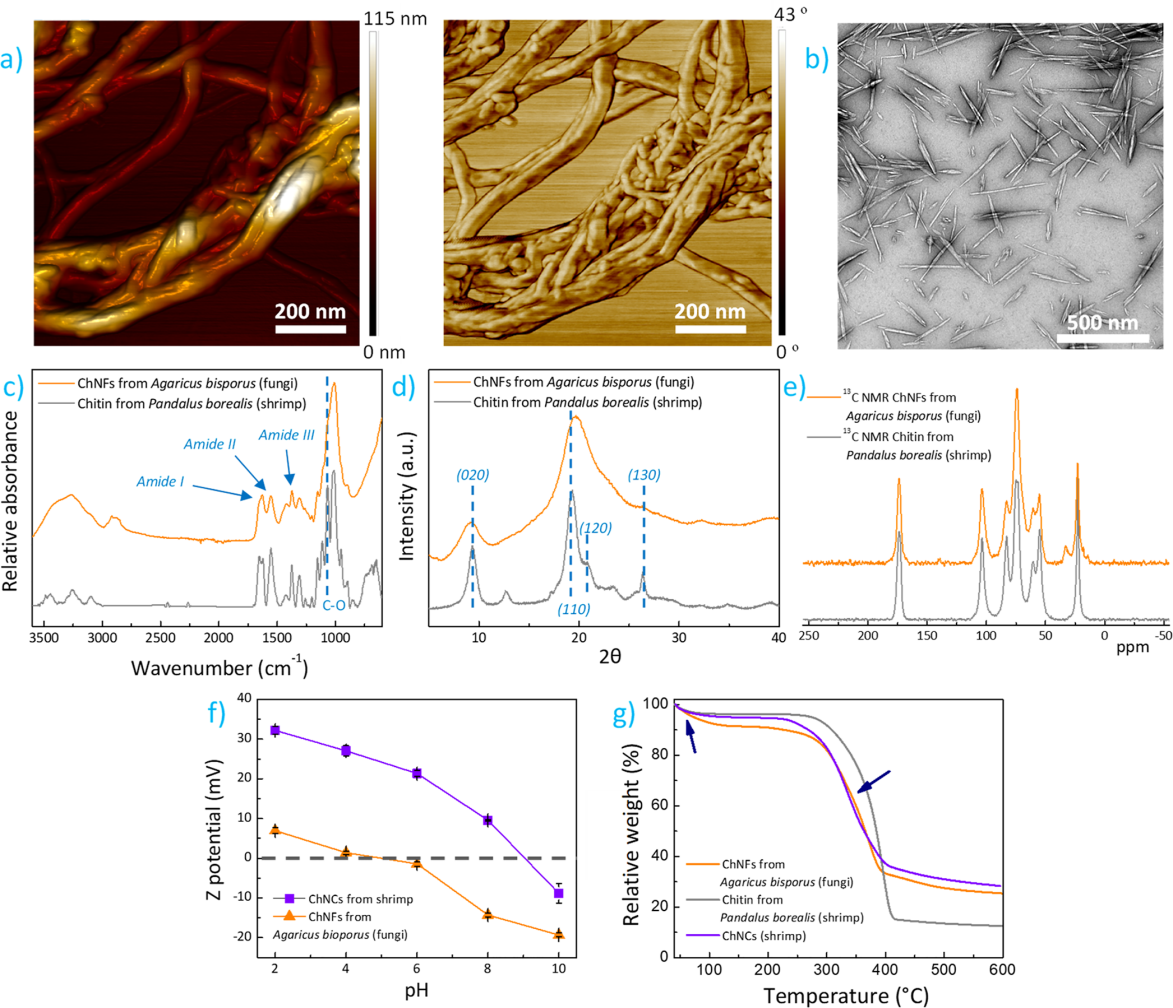
Morphological and conformational characterization: (a) contact-mode AFM height and phase images of isolated ChNFs from *Agaricus bisporus*; (b) transmission electron microscopy image showing isolated ChNCs from commercial α-chitin powder under HCl treatment; (c) ATR-FTIR spectra of ChNFs and α-chitin powder; (d) XRD patterns of ChNFs and α-chitin powder; (e) ^13^C NMR spectra of ChNFs and α-chitin powder; (f) Z-potential of ChNF and ChNC aqueous dispersions at different pH values; (g) thermogravimetric curves corresponding to ChNF from *Agaricus bisporus*, crustacean α-chitin, and crustacean ChNCs.

The morphological features of ChNFs were first characterized by microscopy. A fibrillar-like material with diameters in the range of few nanometers is observed in the contact-mode AFM height and phase images shown in Figure 2a. Such morphology resembles the one produced by mechanically or enzymatically processing cellulose to obtain CNFs, which has a low degree of fibrillation and yields bundles with diameters of *ca*. 20 nm.^38^ A more detailed morphological observation by transmission electron microscopy (TEM) in Figure S3 reveals that ChNFs reach lengths expanding up to ∼1 μm, which is larger than the diameter and length over single α-chitin crystallites (average values of 2–5 nm and ∼300 nm, respectively).^13^ These observations suggest that obtained fibrillary material is composed upon the aggregation of several α-chitin crystallites.^18,39^ For comparison, ChNCs were isolated from α-chitin by an acid hydrolysis process assisted by tip sonication. A 3 M HCl solution at 85 °C for 90 min hydrolyzes the glycosidic bonds of chitin, while a tip-sonication step renders colloidal chitin.^32^ This process has been selected given its simplicity, and potentially lower environmental footprint over other chemically intensive and time-consuming processes (up to 5 M HCl, 104 °C under reflux or reactions times of 18 h).^14,40,41^ As demonstrated by the TEM micrograph in Figure 2c, rod-shaped chitin nanoparticles are obtained (50–300 nm in length, 8–15 nm in width),^9^ which agrees with literature.^14^

Attenuated total reflectance-Fourier transform infrared spectroscopy (ATR-FTIR) and X-ray diffraction (XRD) in Figure 2c and d, respectively, provide additional information about the isolated material. For comparison, data corresponding to commercially available purified chitin powder isolated from *Pandalus borealis* shrimp are also shown. ChNFs present the characteristic absorption bands of chitin, with the broad band at 3650–3200 cm^–1^ due to the −OH stretching, the −CH bands at 2911 and 2841 cm^–1^, the amide I, II, and III bands at 1628, 1556, and 1315 cm^–1^, respectively, and the sharp peaks at 1378 and 1029 cm^–1^ due to the CH_3_ symmetrical deformation and C–O–C groups in chitin, respectively.^42,19^ The amide III band confirms the presence of chitin instead of chitosan. However, the lower intensity of the amide bands in comparison with raw chitin suggest the presence of an additional phase, which according to literature is identified as β-glucans that remain covalently bonded to nanofibrils.^19^ As β-d-glucans are polysaccharides composed of d-glucose monomers linked by β-glycosidic bonds and do not contain nitrogen, the glucosamine content (or chitin content) in the isolated chitin nanofibril–glucan complexes can be estimated from CHN elemental analysis (carbon, hydrogen, nitrogen) by multiplying by 14.199 nitrogen content.^43^ With a nitrogen content of 3.07 ± 0.09 wt % (well below the theoretical 6.89 wt % nitrogen found in chitin), a chitin content of 43.6% is obtained, being the remaining material mostly composed by glucans.^19^

The XRD pattern of ChNFs indicated the occurrence of a semicrystalline material, where two well distinguishable crystalline peaks at 2θ = 9.2 and 19.7° corresponding to (020) and (110) planes of chitin are seen, correlating well with high degrees of *N*-acetylation.^44^ The narrower diffraction peaks (note also the weaker 2θ = 20.5 and 26.2°) match with the crystalline α-chitin form,^42^ while the large halo indicates the presence of an amorphous material such as β-glucans.^18^ The crystallinity index for ChNFs can be obtained from the (020) reflection as
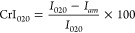
1Crystallinity values of 89.4% and 59.1% are obtained for α-chitin and ChNFs, respectively. Such decreased crystallinity does not necessarily correlates with a lower *N*-acetylation (DA) values for pure samples,^44^ because the contribution of amorphous glucans should be also considered. Besides, the interplanar spacing (*d*) between adjacent planes having (020) Miller indices can provide valuable information on the conformational features of the chitin. The *d* can be obtained from the diffraction patterns according to the Bragg’s law as
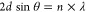
2where θ is the diffraction angle, *n* is an integer, and λ is the wavelength of the radiation used. *d*_020_ values of 9.50 and 9.68 Å are obtained for pure α-chitin and fungal ChNFs, respectively. To gain perspective, *d*_020_ values of 9.70–9.78 Å have been reported for chitin microcrystals extracted from Antarctic krill (*Euphausia superba*), crab, and shrimp shells upon HCl hydrolysis.^45^ This larger spacing for ChNCs obtained after HCl treatment results from the harsh demineralization and deproteination treatments needed to extract chitin from crustacean exoskeletons.^17^ Although the increased interplanar spacing suggests an expansion of the crystal lattice induced by a reduced DA, one must consider that chitosan with a DA as low as 7.2% presents a *d*_020_ value of 7.42 Å.^44^ Therefore, XRD results suggest that the mild isolation process here applied (short reaction times, low temperatures, low basicity) renders α-chitin with a high DA where the hydrogen bonding within the chitin crystallites is not disrupted, together with amorphous glucans.

As one of the most accurate and reproducible technique for the degree of DA estimation, solid-state carbon-13 nuclear magnetic resonance (^13^C NMR) analyses were conducted and the results are shown in Figure 2e (enlarged ^13^C NMR spectra and the corresponding chemical shifts are shown in Figure S4 and Table 1, respectively).^46^ The DA was estimated from the integral of methyl carbon divided by the summation integrals of carbon atoms of the d-glucopyranosyl ring as^47^
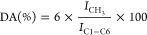
3where *I*_CH_3__ accounts for the integral of the methyl peak and *I*_C1–C6_ considers all the carbon groups in the backbone, respectively. Both spectra are dominated by the features ascribed to chitin, with the C=O signal at 174 ppm, the C-2 at 55 ppm, and the CH_3_ at 23 ppm. Besides, C-4 and C-6 carbon signals do not show doublets, confirming the occurrence of chitin rather than chitosan.^47,48^ The marked intensity of the CH_3_ signals for both samples suggest a high DA as opposed to chitosan, which shows low intensities for this signal. In fact, DA values of 94.1% and 75.8% are obtained according to eq 3, indicating the successful isolation of chitin from fungi. The slight asymmetry of the C-1 peak for ChNFs (signal occurring at 104 and 102 ppm for chitin and β-d-glucan, respectively),^49^ the new signal at 33 ppm and the shoulder appearing close to the C-3 signal (at lower ppm values) is indicative of (1 → 3)-β-d-glucans that remain covalently linked to chitin through a carbonyl linkage.^47^

**Table 1:** Chemical Shifts (δ, ppm) for Chitin Samples Obtained by ^13^C NMR

	signal (ppm)							
sample	C=O	C1	C4	C5	C3	C6	C2	CH_3_
α-chitin^a^	173.8	104.1	83.0	75.7	73.3	60.8	55.2	22.8
α-chitin	173.7	103.6	82.8	74.9	73.3	60.5	54.9	22.6
ChNFs	173.7	103.6	82.9	74.1	74.1	60.5	55.1	22.8

a α-Chitin from ref (47).

As nanoparticle surface charge mediates cell–material interactions,^50^ the surface charge of water-dispersed biocolloids at varying pH values was measured, and the results are shown in Figure 2f. At low pH values, ChNFs show a positive net charge due to protonation of the *N*-acetyl groups of chitin. The surface becomes negatively charged as the media becomes alkaline to reach −20.2 mV at pH 10, with an isoelectric point around pH 4.5. Obtained charges are more negative than the results obtained for ChNCs (see Figure S5 for ATR-FTIR and XRD results characteristic of α-chitin), where a net positive charge of +32 mV at pH = 2 with an isolelectric point at pH 9.1 is seen. The more negative charge of ChNFs over ChNCs at a given pH suggests a lower fraction of amine groups available to undergo deprotonation. This lower charge induces electrostatic repulsion forces among nanoparticles to improve their colloidal stability, similar to what has been observed for CNCs.^51^ Finally, Figure 2g shows the thermogravimetric analysis (TGA) curves of a ChNF film, α-chitin powder, and ChNCs. Overall, a very similar thermal stability of ChNFs and ChNCs is observed. An initial weight loss centered at 80 °C corresponding to adsorbed water evaporation (blue arrow), together with a wide and marked thermodegradation event occurring in the 280–405 °C range originating from the degradation of 2-amino-2-deoxy-d-glucopyranose units in chitin is observed.^52^ ChNFs adsorb more water (by weight) in comparison with chitin powder, and the degradation curve becomes wider for ChNFs. Besides, a char equivalent of ∼25 wt % is obtained at 650 °C for ChNFs. It is worthy to note that ChNFs show an improved resistance toward thermodegradation over the ubiquitous CNCs extracted through sulfuric acid hydrolysis.^53^

**Figure 3:**
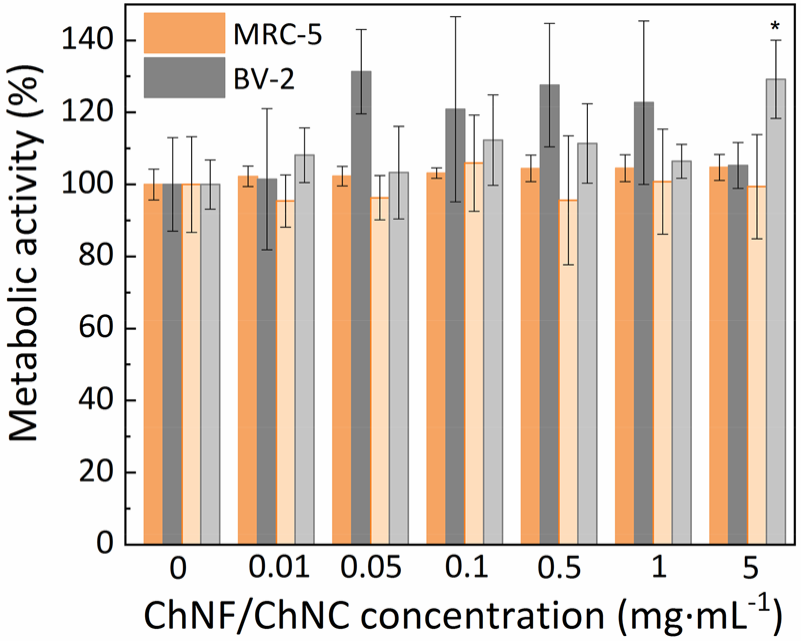
Metabolic activity of MRC-5 and BV-2 cells in the presence of increasing concentrations of ChNFs (dark orange and dark gray) or ChNCs (light orange and light gray) after 24 h. Asterisks (*) indicate significant differences (*p* < 0.05) with respect to the control (absence of ChNFs or ChNCs; *n* = 5).

To evaluate the potential cytotoxicity of the isolated ChNFs, the metabolic activity of two different cell lines was quantified by means of an AlamarBlue assay (Figure 3). After 24 h in contact with increasing concentrations of ChNFs (from 0 to 5 mg·mL^–1^), no statistically significant differences (*p* < 0.05) were observed on the metabolic activity of human lung fibroblasts (MRC-5) with respect to the control (i.e., cells in the absence of ChNFs). Similarly, the metabolic activity of murine microglia (BV-2) was always similar or slightly higher than that observed in the control, confirming again the cytocompatibility of the ChNFs isolated from fungi at the studied concentrations. In the presence of ChNCs, both cell lines showed a similar behavior, and statistically significant differences were only observed in BV-2 cells exposed to the highest concentrations (i.e., 5 mg·mL^–1^) of ChNCs. It should be noted that the cytotoxicity of novel materials at the nanoscale based on natural biopolymers strongly depends on the source, isolation protocol, functionalization, dimensions and studied cell line.^26,37^ Thus, a direct comparison between different nanomaterials is difficult due to the lack of well-established protocols. Some review papers have tried to summarize those recent studies dealing with the cytotoxicity of cellulose-based nanomaterials, which share structural and chemical similarities to the ChNFs presented herein.^54,55^ Hanif et al. prepared cellulose nanocrystals of controlled shape and size and studied their potential cytotoxicity with murine fibroblasts (NIH3T3).^28^ The prepared CNCs did not have any detrimental effect on cell viability at concentrations of up to 250 μg·mL^–1^. However, cell viabilities below 80% were recorded for higher CNCs concentrations (i.e., 500 and 1000 μg·mL^–1^). In a similar study, Pereira et al. explored the cytotoxicity of CNFs derived from cotton on bovine fibroblasts.^56^ As determined by flow cytometry, CNFs did not impact cell viability for concentrations of up to 200 μg·mL^–1^. Nonetheless, a dose-dependent cytotoxicity was observed for higher concentrations with cell viabilities of 72 and 37% for concentrations of 1 and 5 mg·mL^–1^, respectively. In view of our results, it remains feasible to argue that the ChNFs isolated herein are comparatively safer than previously reported nanomaterials derived from naturally sourced polymers and show a behavior similar to that of the ChNCs obtained via the traditional acid hydrolysis from chitin powder from shrimps.

**Figure 4:**
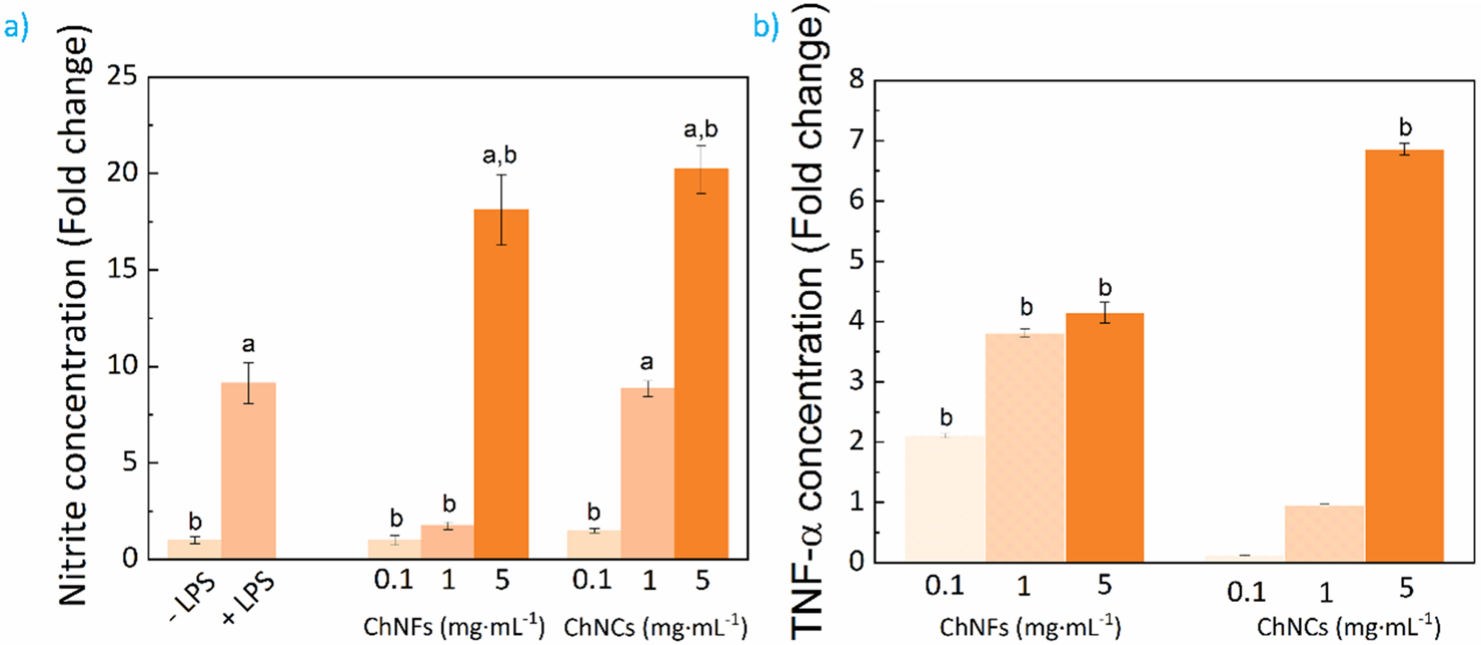
(a) Fold change with respect to the negative control (i.e., cells in the absence of ChNFs or ChNCs) of nitrite concentration in the supernatant of BV-2 cells exposed to different concentrations of ChNFs and ChNCs for 24 h. (b) Fold change with respect to the positive control (i.e., cells stimulated with 20 ng·mL^–1^ of LPS) of TNF-α concentration in the supernatant of BV-2 cells exposed to different concentrations of ChNFs and ChNCs for 24 h. “a” and “b” indicate significant differences (*p* < 0.05) with respect to the negative and positive control, respectively (*n* = 3).

Despite the positive results observed for dispersed ChNFs in terms of cytotoxicity, where the metabolic activity of both MRC-5 and BV-2 cells remained unaltered for a wide range of ChNF concentrations, deeper biological tests are required to ensure safe use and explore future applications of this biocolloid. Herein, the secretion of inflammatory mediators by BV-2 cells at varying ChNF concentrations was investigated. BV-2 is murine microglia that play a major role in the regulation of neuroinflammatory processes and are quickly activated in response to exogenous insults, including nanomaterials. Thus, they have been previously explored as a cellular model to study the inflammatory response to various nanoparticles (e.g., silver and titanium dioxide nanoparticles).^30,57^ As observed in Figure 4a, the concentration of nitrites in the supernatant of BV-2 cells gradually increased with the ChNF concentration. At a ChNF concentration of 1 mg·mL^–1^, despite no statistically significant differences (*p* < 0.05) being observed, the presence of nitrites was 1.7 times higher than for the negative control (i.e., cells in the absence of ChNFs), but still significantly lower than the concentration observed in the positive control (i.e., cells stimulated with 20 ng·mL^–1^ of LPS). When increasing the ChNF concentration up to 5 mg·mL^–1^, the concentration of nitrites was 18.1 times higher than for the negative control and 2.0 times higher than for the positive control, being these differences statistically significant (*p* < 0.05). In the case of ChNCs, the concentration of nitrites at a ChNC concentration of 1 mg·mL^–1^ was significantly higher (*p* < 0.05; 8.8 times higher) than the one observed in the negative control, being similar to the levels observed in the positive control. At the highest concentration of ChNCs (i.e., 5 mg·mL^–1^), the release of nitrites was 20.2 times higher than for the negative control and 2.0 times higher than for the positive control, being these differences statistically significant (*p* < 0.05). The measurement of nitrites is regularly used to estimate the production of nitric oxide by cells, which is a pro-inflammatory mediator. As a complementary assay, the secretion of tumor necrosis factor (TNF-α), which represents an inflammatory cytokine, by BV-2 cells in the presence of ChNFs was further studied (Figure 4b). BV-2 cells secreted negligible levels of TNF-α in the absence of ChNFs (<100 pg·mL^–1^), whereas they secreted >4000 pg·mL^–1^ when stimulated with 20 ng·mL^–1^ (i.e., positive control), thus validating our *in vitro* inflammation model. BV-2 cells secreted higher levels of TNF-α as the concentration of ChNFs increased, being always significantly (*p* < 0.05) higher than in the case of the positive control. In the case of ChNCs, the secretion of TNF-α was similar to the positive control at a concentration of 1 mg·mL^–1^. At a ChNC concentration of 5 mg·mL^–1^, the concentration of TNF-α in the cell supernatant was 6.9× higher than the one observed in the positive control. Taken together, these results suggest a dose-dependent pro-inflammatory response of microglia to both ChNFs and ChNCs.

Obtained results are in agreement with previous *in vitro* and *in vivo* observations dealing with the inflammatory response induced by biobased colloids. As for cytotoxicity studies, there exists a strong interplay between the size, shape, surface functionalities, raw material source, preparation procedure, and inflammatory response induced by the resulting nanomaterials, making a direct comparison between different studies challenging. For example, Menas et al. concluded that CNCs caused a more severe inflammatory response on human lung epithelial cells than nanofibrillated cellulose.^58^ In a different study, a cationic derivative of cellulose nanocrystals also induced a pro-inflammatory response on murine macrophages, being the response dependent on the surface functionalities.^59^ These *in vitro* observations are further supported by *in vivo* results, where CNCs induce pulmonary toxicity in mice by eliciting oxidative stress, tissue damage and a robust inflammatory response.^60^ Considering the growing use of emerging biocolloids and their potential technological applications,^7,13^ it results vital to conduct detailed biological evaluations of these biocolloids to gain more insights about the particular interaction of these nanomaterials and cells, tissues and organs. In the present preliminary study, ChNFs were challenged with BV-2 at relatively high concentrations (>100 μg·mL^–1^) to study their potential pro-inflammatory effect. However, we estimate further *in vitro* studies are required with the aim of evaluating the effect of processing, surface functionalities, and morphological aspects of the ChNFs isolated herein on cell behavior.

### Toxicity and Cell Proliferation onto Chitin Nanofibril Films

**Figure 5:**
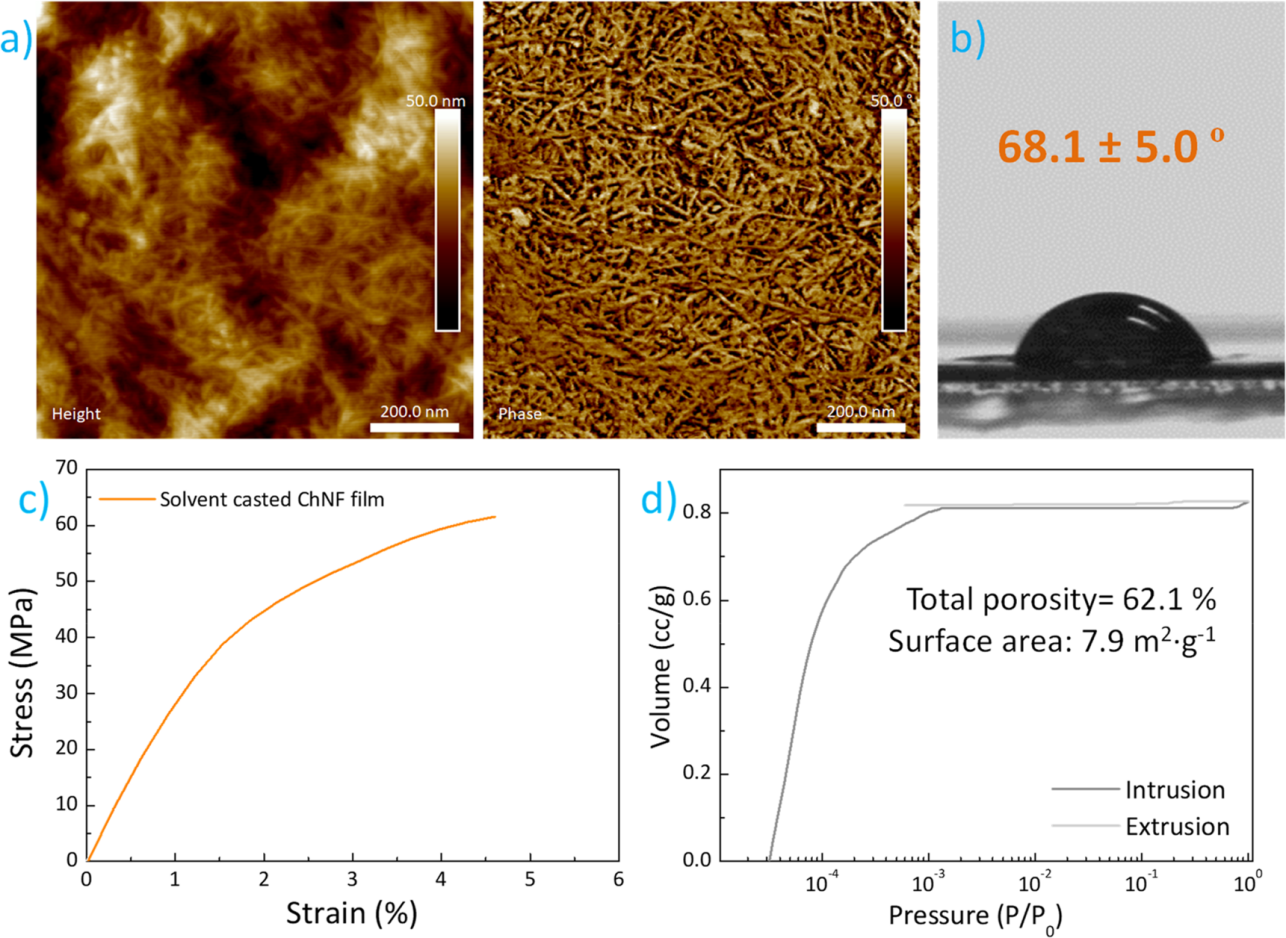
Solvent-casted ChNF film characterization: (a) surface morphology as revealed by tapping-mode AFM height and phase images; (b) representative image of a water drop at the surface of a ChNF film; (c) representative uniaxial tensile stress–strain test; and (d) intrusion/extrusion curves of mercury-porosimetry.

The surface characteristics of materials play a determinant role in the resulting cell/material interactions.^61,62^ Accordingly, the surface morphology of solvent-casted ChNF films has been investigated by tapping-mode AFM. Figure 5a shows the height and phase AFM images of a free-standing ChNF film. A relatively flat surface composed of overlapped nanofibrils with a random in plane orientation is observed. The achieved network structure originates from the structural flexibility and high aspect-ratio of ChNFs, which show a tendency toward physical entanglement during water evaporation. As the nanofibrils remain covered by an amorphous layer (glucans), individual nanofibrils are difficult to observe in the images (higher-magnification AFM height and phase images in Figure S6 suggest a ChNF width of approximately 7 to 17 nm). The surface roughness of the films, determined by root-mean-square roughness (*R*_q_) and mean roughness (*R*_a_) parameters reaches 19.3 and 15.6 nm, respectively.^63^ Such low values denote the formation of highly smooth surfaces upon the solvent-casting of ChNF aqueous dispersions. The surface wettability was assessed by water contact angle measurements. A contact angle of 68.1 ± 5° is seen in Figure 5b for a 5 μL water drop onto a ChNF film, indicating a predominantly hydrophilic nature as occurring with nanocellulose films.^63^ In spite of being hydrophilic, ChNF films present a good resistance to break or redispersion when immersed in distilled water (Figure S2), offering an undeniable advantage toward biomedical or packaging applications over their nanocellulose analogues, whose water stability is usually poor.^64^ This stability originates from the synergy between glucans and nanofibrils,^18^ and contrast with the swelling and subsequent nanoparticle dispersion suffered by CNC-based nanopapers when in contact with aqueous systems.

Besides, the mechanical and morphological features of ChNF films were investigated before conducting biological tests. The tensile properties of ChNF films were determined according to uniaxial tests and the representative stress–strain curve is shown in Figure 5c. The ChNF film presents a brittle behavior whose Young’s modulus (*E*) reaches 3415 MPa, a maximum tensile stress (σ_*y*_) of 61.5 MPa and an elongation at break (ε_b_) of 4.6%. It should be noted that, in spite of the processes simplicity do not requiring filtration and a subsequent hot-pressing,^65^ the observed modulus and ultimate strength values remain comparable to the results reported for the majority of biodegradable thermoplastic materials.^66,67^ This may be due to the combination of the physical entanglement and secondary attraction forces of individual nanofibers, together with the native amorphous glucans among nanofibrils improving the binding of the whole material as naturally occurs in the fungal cell walls.^18^ Besides, an enhanced ductility in comparison with nanopapers based on CNCs (ε_b_ = 1.9 ± 0.2%),^63^ certain CNF-based films (ε_b_ = 2.1 to 10.1%, depending on the cellulose origin and film porosity),^68^ bacterial cellulose (ε_b_ = 2.4 ± 0.3%),^69^ or crustacean-derived HCl-hydrolyzed chitin nanowhisker films (ε_b_ = 1.2 ± 0.4%)^70^ is achieved. However, a lower modulus and tensile strength have been obtained in comparison with the ChNF film by Nawawi et al. (6.9 GPa, 204 MPa, respectively).^18^ The largest load-resistance shown by Nawawi et al. can be explained by a reduced porosity and enhanced secondary attraction forces between nanofibrils achieved upon filtration and subsequent pressing in an oven at 120 °C for 3 h under 5 kg weight.^18^ For further verification, we conducted a mercury intrusion porosimetry analysis to investigate the pore characteristics of the ChNF films. As seen in Figure 5d, a porosity of 62.1% and film surface area of 7.9 m^2^·g^–1^ was obtained (similarly to nanocellulose films fabricated by solvent casting; porosity = 56%, surface area = 11.0 m^2^·g^–1^).^71^ The abundant pores within the interior of the film can act as crack initiation sites and lead to a material embrittlement effect when subjected to external tensile stresses.^72^ A filtration approach could be explored in the future to reduce film porosity and improve the fracture properties of the ChNF films. This porosity, despite having a detrimental effect on strength-related properties, may be beneficial for the use of this material as a scaffold for tissue engineering applications since it facilitates the diffusion of nutrients and oxygen. It should be noted that a solvent-casting approach was followed here to keep the native film surface morphology intact and avoid undesired patterning of the ChNF film by the molds used during hot pressing.

**Figure 6:**
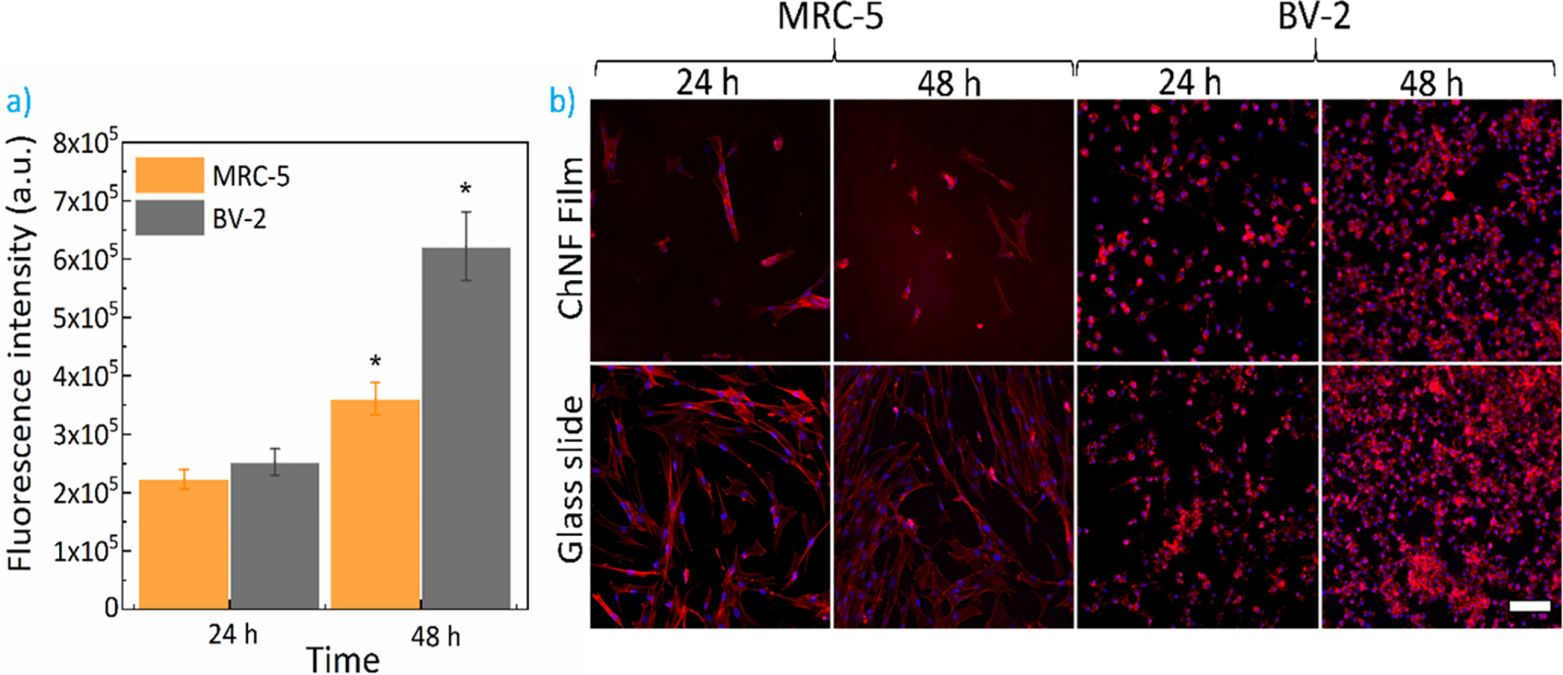
(a) Metabolic activity of MRC-5 and BV-2 cells onto ChNF films and their (b) corresponding fluorescent micrographs (Blue-Dapi-Nuclei; Red-Rhodamine phalloidin-actin filaments). Scale bar: 100 μm. Asterisks (*) indicate significant differences (*p* < 0.05) with respect to the control (fluorescent intensity of cells at 24 h; *n* = 6).

Obtained fibrillary-like surface morphology and mechanical properties, in combination with the noncytotoxicity observed for water-dispersed nanofibrils, make ChNF films potential candidates for biomedical uses. An additional attractive originates from its biobased character, which enables environmentally sustainable biomedical materials as opposed to current conventional choices relying on petroleum-derived polymers, such as poly(vinylidene fluoride) or poly(ε-caprolactone).^73^ Therefore, the potential of ChNF films to allow cell growth and adhesion was also evaluated with MRC-5 and BV-2 cells. As observed in Figure 6a, the metabolic activity of both MRC-5 and BV-2 cells increased with time, confirming that cells were able to grow in the presence of the ChNF films. Accordingly, the calculated metabolic activities at 48 h were 1.6× and 2.5× higher than those at 24 h for MRC-5 and BV-2 cells, respectively. As concluded from the fluorescent micrographs in Figure 6b, very few MRC-5 cells were observed on the ChNF films in comparison to the control (i.e., glass slide), suggesting a poor interaction between the cells and the biomaterial. This could be ascribed to the envisaged structure of our ChNFs, that may contain hydrophobins (i.e., cysteine-rich proteins found in filamentous fungi and mushrooms),^20^ thus limiting the adhesion of human fibroblasts, as previously reported.^74,75^ In contrast, BV-2 cells were able to adhere and grow on the ChNF films, showing morphologies comparable to those of the cells observed on the glass slide. We hypothesize that the activation of microglia by the presence of ChNFs (as demonstrated in Figure 4) may increase the expression of integrins, thus facilitating cell adhesion to the biomaterial. Previous studies have demonstrated that the activation of microglia through the stimulation with proinflammatory cytokines (e.g., TNF-α, IFN-α, etc.) or LPS significantly increases the adhesion of these cells to otherwise poorly adherent substrates (e.g., laminin) via the expression of integrins,^76,77^ which supports our hypothesis.

The relatively low purity (presence of 56 wt % glucans) of fungi-derived ChNFs over crustacean-derived ChNCs originates from the combination of the inherent presence of glucans polysaccharide in fungi together with the mild isolation process applied. In spite of such a lower purity, ChNFs offer clear advantages regarding environmental sustainability and scale-up potential. In fact, and as opposed to its crustacean-derived analogues, fungal nanochitin can be certainly upscaled following the 12 principles of green chemistry.^78^ Mechanical blending, filtration, and alkaline deproteinization are easily scalable processes as they are currently applied at the industrial level. Besides, the use of renewable feedstock together with the short and mild deproteinization (low temperature, ambient pressure) steps needed ensure minimal toxicity to human health and the environment with low energy demand. Areas for improvement in the near future include the optimization of NaOH concentration and yield increase, so the use of materials is maximized (atom economy). As such, we anticipate that the near future could witness the industrial production of fungal nanochitin for its exploitation in a variety of applications (whether biomedical or not) that bear the benefit of a noncytotoxic and are comparatively safer character than other biocolloid analogues.

## Conclusions

This work represents the first evaluation of the cytotoxicity and inflammatory effects of chitin nanofibrils isolated from mushrooms. Chitin nanofibrils with diameters in the range of a few nanometers and lengths extending nearly 1 μm are isolated from mushroom under mild conditions. AFM, TEM, ATR-FTIR, XRD, ^13^C NMR and elemental analysis results indicate that these nanofibrils are composed upon the aggregation of several α-chitin crystallites surrounded by amorphous β-glucans. Precisely, isolated ChNFs present a crystallinity degree of 59.1% and are composed by semicrystalline chitin where β-glucans remain covalently bonded to nanofibrils (44 wt % chitin). According to ^13^C NMR, the chitin fraction presents a *N*-acetylation degree of 75.8%. The metabolic activity of human lung fibroblasts and murine microglia for colloidally stable ChNF aqueous dispersions confirms a good cytocompatibility for concentrations as large as 5 mg·mL^–1^. Free-standing chitin nanofibril films were then fabricated by a simple solvent-casting approach. In spite of the high film porosity of 62.1%, as indicated by mercury intrusion porosimetry, a Young’s modulus of 3415 MPa and an ultimate strength of 61.5 MPa were achieved thanks to the combination of the physical entanglement and secondary attraction forces of individual nanofibers, together with native amorphous glucans improving the material binding as naturally occurs in the fungal cell walls. AFM observations reveal the formation of smooth films composed of a homogeneous structure of closely packed nanofibrils with a random in plane orientation that remain covered by amorphous glucans. The obtained films are hydrophilic as indicated by a water contact angle value of 68.1 ± 5.0° and allow the growth of human fibroblasts and murine microglia. While poor adhesion is observed between fibroblasts and ChNF films, microglia can adhere and grow on the surface of the biomaterial. This behavior is ascribed to the activation of microglia in the presence of ChNFs and the corresponding expression of integrins.

Altogether, these results highlight a comparatively safer character of fungal-derived chitin nanofibrils over analogous biocolloids such as cellulose nanocrystals and cellulose nanofibrils. Thereby, ChNFs from fungi emerge as potential candidates for environmentally sustainable biomedical materials in contraposition with conventional biomedical thermoplastics based on petroleum-derived polymers such as poly(vinylidene fluoride) or poly(ε-caprolactone).

## Data Availability

The data supporting this work is shown in the Supporting Information.

## Supporting Information Available

Optical photographs showing ChNF colloidal dispersion stability in water and ChNF-film stability when immersed in water; High magnification TEM image of ChNFs, ^13^C NMR of ChNFs and α-chitin, and ATR-FTIR/XRD characterization for ChNCs; High-magnification AFM images of a solvent-casted ChNF film (PDF)
