# Supplementary material for: Cytotoxicity and Inflammatory Effects of Chitin Nanofibrils Isolated from Fungi
Source: Biomacromolecules. 2023 Nov 21;24(12):5737–48. doi: 10.1021/acs.biomac.3c00710 (PMC10716858; doi:10.1021/acs.biomac.3c00710)
Supplement: Supplementary file 1 — bm3c00710_si_001.pdf [file bm3c00710_si_001.pdf]

Supporting Information

Cytotoxicity and Inflammatory Effects of Chitin Nanofibrils Isolated from Fungi

Aitor Larrañaga,[†] *Carlos Bello-Álvarez,* [†] *Erlantz Lizundia,* [‡,§]*

[†] Department of Mining-Metallurgy Engineering and Materials Science, POLYMAT, Faculty of Engineering in Bilbao. University of the Basque Country (UPV/EHU), Plaza Ingeniero Torres Quevedo 1, 48013 Bilbao, Biscay, Spain.

[‡] Life Cycle Thinking Group, Department of Graphic Design and Engineering Projects. University of the Basque Country (UPV/EHU), Plaza Ingeniero Torres Quevedo 1, 48013 Bilbao, Biscay, Spain.

[δ] BCMaterials, Basque Center for Materials, Applications and Nanostructures, Edif. Martina Casiano, Pl. 3 Parque Científico UPV/EHU Barrio Sarriena, 48940 Leioa, Biscay, Spain.

*: Corresponding author: erlantz.liizundia@ehu.eus

Number of pages: 4

Number of tables: 0

Number of figures: 6

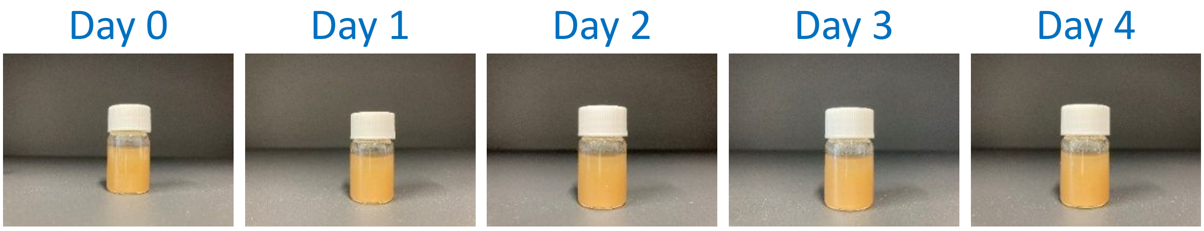

Figure S1. Photographs showing the ChNF aqueous dispersion stability for different periods.

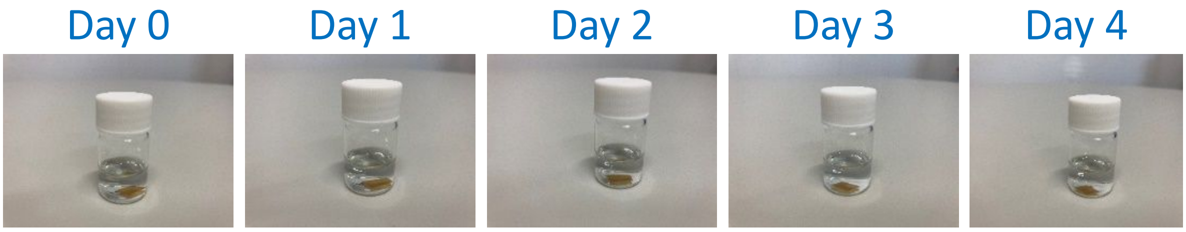

Figure S2. Photographs of as-casted ChNF freestanding films (10 x 10 mm) immersed in distilled water at room temperature for different periods.

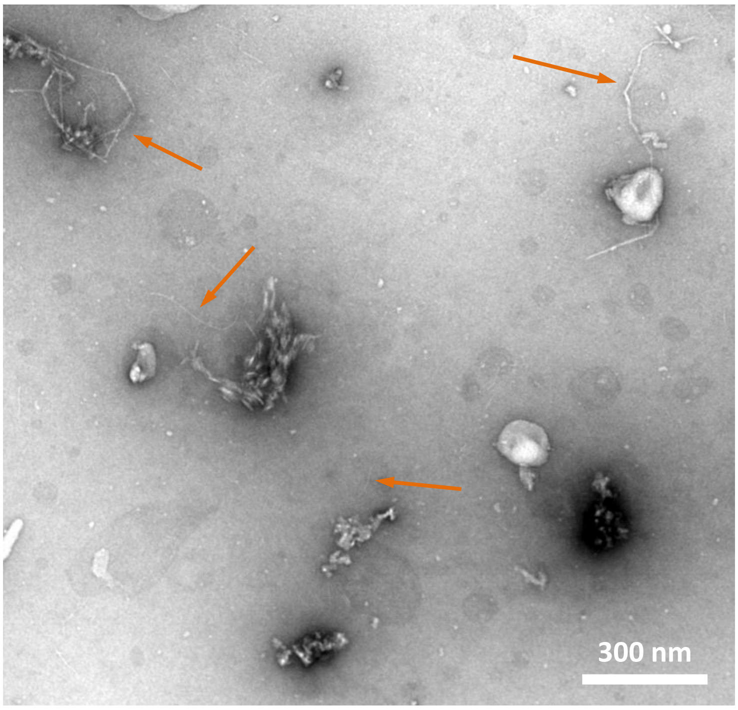

Figure S3. High magnification transmission electron microscopy (TEM) image showing isolated ChNFs from *Agaricus bisporus*. Arrows indicate the occurrence of nanofibrils.

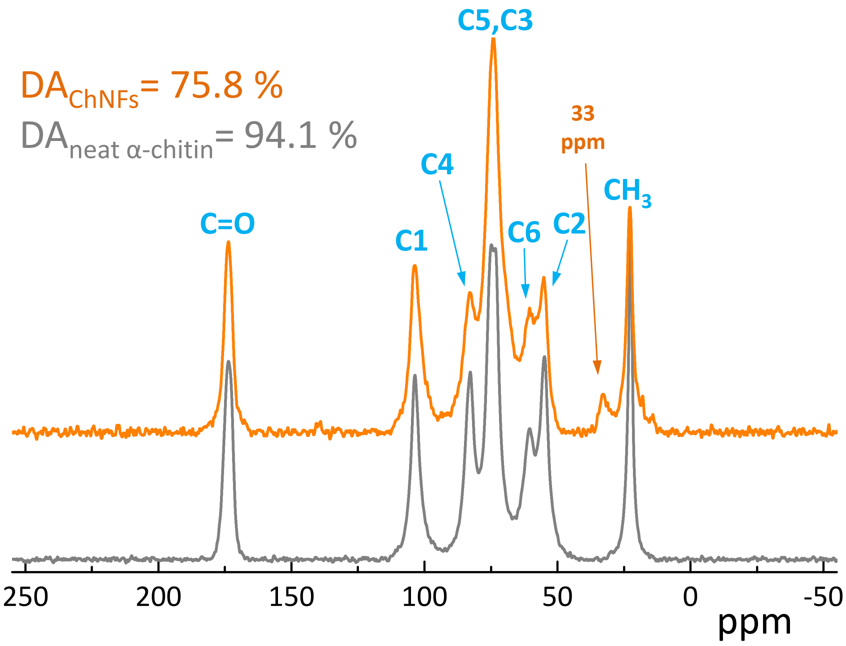

Figure S4. Detailed ^{13}C NMR spectra of isolated ChNFs (orange line) and commercial neat α-chitin (grey line). Assigned carbons are highlighted together with the estimated degree of acetylation values.

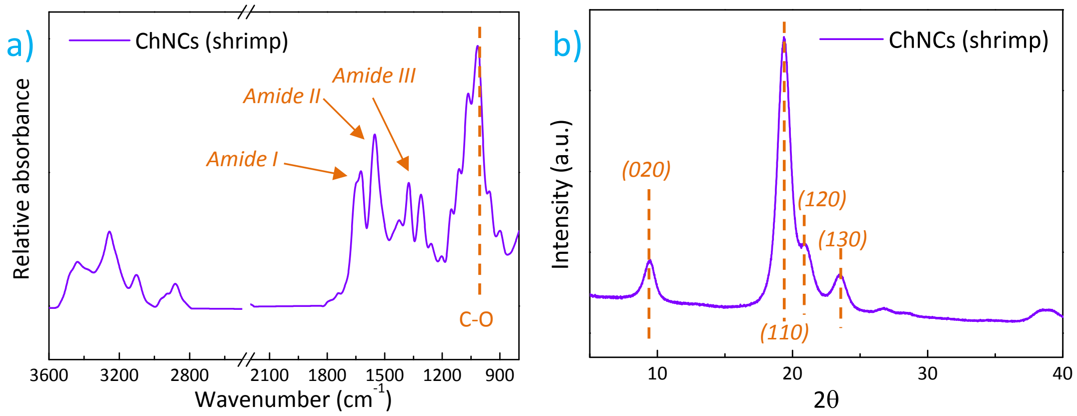

Figure S5. Chitin nanocrystals (ChNCs) characterization: a) ATR-FTIR spectrum and b) XRD pattern.

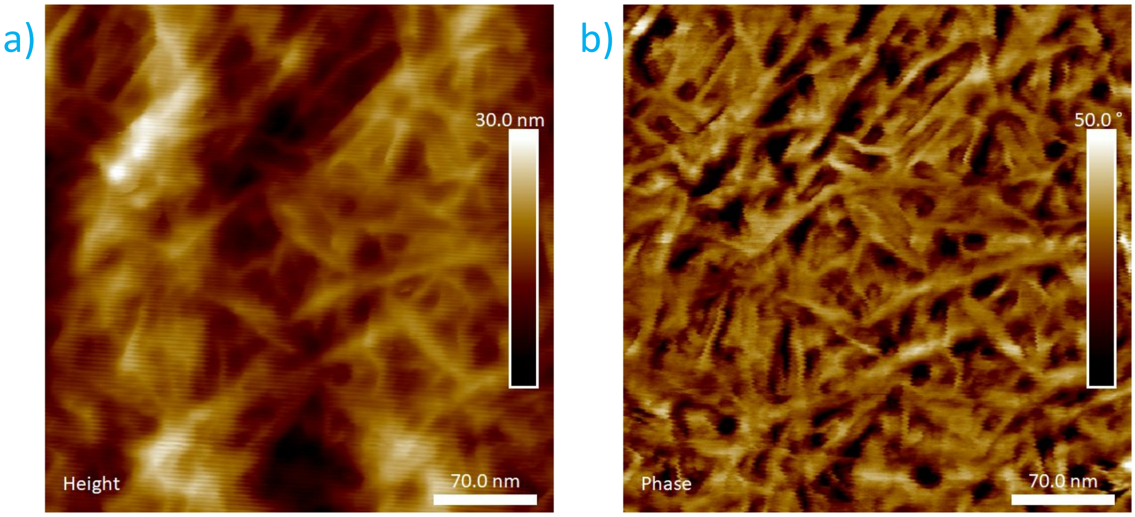

Figure S6. High magnification tapping-mode AFM height (a) and phase (b) images of a solvent-casted freestanding ChNF film.
